# Content and Bioaccessibility of Minerals and Proteins in Fish-Bone Containing Side-Streams from Seafood Industries

**DOI:** 10.3390/md22040162

**Published:** 2024-04-03

**Authors:** Marie Bagge Jensen, Jette Jakobsen, Charlotte Jacobsen, Jens J. Sloth, Jone Ibarruri, Carlos Bald, Bruno Iñarra, Niels Bøknæs, Ann-Dorit Moltke Sørensen

**Affiliations:** 1 DTU Food, National Food Institute, Technical University of Denmark, 2800 Kongens Lyngby, Denmark; jeja@food.dtu.dk (J.J.); jjsl@food.dtu.dk (J.J.S.); adms@food.dtu.dk (A.-D.M.S.); 2 AZTI, Food Research, Basque Research and Technology Alliance (BRTA), Parque Tecnológico de Bizkaia, Astondo Bidea, Edificio 609, 48160 Derio-Bizkaia, Spain; jibarruri@azti.es (J.I.); cbald@azti.es (C.B.); binarra@azti.es (B.I.); 3 Royal Greenland, 9230 Svenstrup, Denmark; nibk@royalgreenland.com

**Keywords:** fish industry, valorisation, undersized hake, cod backbone, salmon backbone, in vitro digestion

## Abstract

With the aim to upcycle fish side-streams, enzymatic hydrolysis is often applied to produce protein hydrolysates with bioactive properties or just as a protein source for food and feed. However, the production of hydrolysates generates a side-stream. For underutilized fish and fish backbone this side-stream will contain fish bones and make it rich in minerals. The aim of this study was to assess the relative bioaccessibility (using the standardized in vitro model INFOGEST 2.0) of minerals in a dietary supplement compared to bone powder generated after enzymatic hydrolysis of three different fish side-streams: undersized whole hake, cod and salmon backbones consisting of insoluble protein and bones. Differences in the bioaccessibility of protein between the powders were also investigated. The enzyme hydrolysis was carried out using different enzymes and hydrolysis conditions for the different fish side-streams. The content and bioaccessibility of protein and the minerals phosphorus (P), calcium (Ca), potassium (K) and magnesium (Mg) were measured to evaluate the potential of the powder as an ingredient in, e.g., dietary supplements. The bone powders contained bioaccessible proteins and minerals. Thus, new side-streams generated from enzymatic hydrolysis can have possible applications in the food sector due to bioaccessible proteins and minerals.

## 1. Introduction

In a world where the population continues to increase, the food sector needs to optimize value chains to increase the utilization of the resources available. This also applies to the seafood sector. In some fish production sites, up to 50–70% of the fish weight currently ends up as side-streams (e.g., backbone, head and viscera, undersized fish). These side-streams are often used as low-value products, e.g., fish meal. However, they contain valuable compounds that may have a nutritionally relevant level of proteins as well as minerals, which could potentially be used for human consumption. An increased utilization of the side-streams will also increase the sustainability of the seafood value chains.

Different potential approaches have been evaluated to increase the utilization degree for human consumption, where many studies have evaluated the production of protein hydrolysates with different functional and bioactive properties, e.g., antimicrobial, antioxidants, flavour enhancer, emulsification, etc. [1,2,3]. To obtain these protein hydrolysates, enzymatic treatment is applied, and a new side-stream is generated: the precipitate after hydrolysis and separation. The composition of the precipitate will vary and depends upon the raw material, where fish backbone will result in a precipitate consisting of insoluble proteins and fish bones [1]. A relative high content of certain minerals in fish bones from different species has been reported [4,5]. The potential utilization of the precipitate generated has received limited attention until now. Due to the content of nutrients (protein and minerals) [1], the precipitate or part of it can potentially find use in food products or as supplements. When substituting one food ingredient or parts of it with a new food ingredient, it is important to assess the nutrient content as well as to evaluate the bioaccessibility of the nutrients of interest in the potential new ingredient. 

The bioaccessibility is the amount of a nutrient which potentially is available for absorption in the intestinal cell [6]. The bioavailability of a compound is said to be dependent on five different factors: the bioaccessibility of the compound, the absorption of the compound by the epithelial cell layer, the distribution of the compound in the body, the metabolism of the compound, and the excretion of the compound [7]. The absorption of minerals occurs in the small intestine via both passive and active transport across the epithelial cell layer. Regardless of the homeostasis mechanism for each of the minerals (calcium (Ca), phosphorus (P), magnesium (Mg) and potassium (K)), they have to be soluble and to exist as free ions in the gastrointestinal tract to become absorbable in the intestinal cell [8,9,10,11,12]. Proteins are absorbed as either free amino acids or as di- and tri-peptides [13]. Different factors—e.g., the composition of the component in the diet—may impact the solubility and thereby the bioaccessibility [5], which is a motivation for investigating the bioaccessibility of the minerals and the proteins present in the bone powder products.

The international standardized in vitro model, INFOGEST 2.0, has successfully been validated for protein hydrolysis in skimmed milk versus an in vivo pig model [14,15]. It is essential for the investigation of mineral bioaccessibility to verify the analytical methods used to quantify the non-bound minerals. 

This study aimed to assess the content and the relative bioaccessibility of the minerals and proteins in bone powder products using the standardised in vitro model INFOGEST 2.0 [14]. In the following, the term “bone powder products” will be used for dried precipitate or sieved fish bones obtained after enzymatic hydrolysis. For the minerals, the focus was on calcium and phosphorus due to their high concentration in fish bones; however, minerals present in lower concentrations (such as magnesium and potassium) were also assessed. Bioavailability of the minerals in the different bone powder products was compared to a calcium supplement. Additionally, the impact of the hydrolysis or heat treatment on the bioaccesibility of minerals and protein in the bone powder was evaluated. The analytical methods used for minerals quantified the total amount of the mineral in the bone powder products and the calcium supplement, as well as the free minerals in the digest from the in vitro model. Protein was measured as nitrogen for all samples. 

## 2. Results

### 2.1. Content of Minerals and Protein in Bone Powder Products and Raw Cod Backbone

The four different bone powder products, the raw cod backbone and a calcium supplement were analysed for their contents of the four minerals magnesium, phosphorus, potassium and calcium. Additionally, the different bone powder products and raw cod backbone were analysed for their protein content. The results are shown in Table 1.

Generally, the bone powder products showed the highest content of calcium, followed by phosphorus, potassium and magnesium (Table 1). HakeE and SalmonE tended to have higher concentrations of certain minerals (calcium and phosphorus) compared to CodE and CodC. It was clear from the results that the bone powder products generated from cod after either enzymatic hydrolysis (CodE) or heating (CodC) were enriched in minerals compared to the original cod backbone (RawCod).

The protein content in the bone powder products ranged from 28% to 54%, while the raw cod product had a content of 19% protein (Table 1). 

### 2.2. Bioaccessibility of Minerals from the Different Bone Powder Products and Raw Cod Backbone

**Figure 1:**
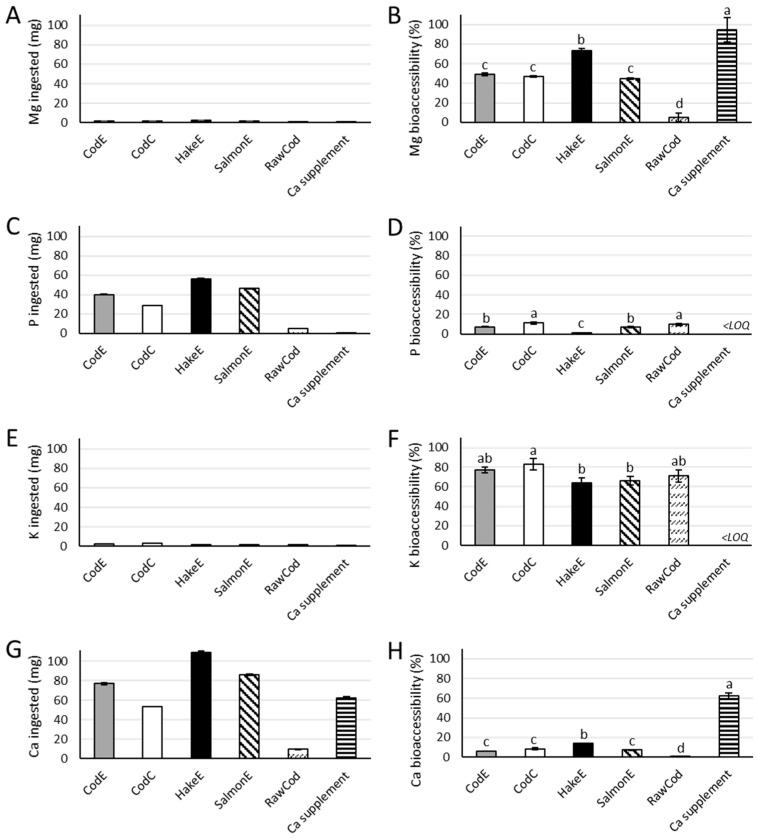
The ingested amounts (mg) of magnesium (Mg) (**A**), phosphorus (P) (**C**), potassium (K) (**E**) and calcium (Ca) (**G**) and their bioaccessibilities (**B**,**D**,**F**,**H**), respectively. All samples were analyzed in triplicate (*n* = 3). Error bars depicts the standard deviation. Different letters indicate significant differences (*p*-value < 0.05). <LOQ = below the limit of quantification. For sample descriptions, refer to Section 4.2.

The bioaccessibility of magnesium, phosphorus, potassium and calcium was then assessed from the different bone powder products using INFOGEST 2.0. The ingested amount of each mineral in the in vitro model and the corresponding bioaccessibility are shown in Figure 1. 

The bone powder products contained low amounts of magnesium and potassium and high amounts of phosphorus and calcium (Table 1). Therefore, there was a difference in the amounts of minerals applied to the in vitro model: low for magnesium and potassium and high for phosphorus and calcium (Figure 1A,C,E,G). In contrast, the bioaccessibilities for magnesium and potassium were higher than for phosphorus and calcium (Figure 1B,D,F,H).

For the different bone powder products, the highest bioaccessbility of magnesium was observed for HakeE, at 74%; meanwhile, no significant differences were observed between the other bone powder products, which varied between 45% and 49% (Figure 1B). The raw cod backbone had a significantly lower bioaccessibility of magnesium (of 5.4%) while the supplement had a bioaccessibility of magnesium of 95%, which is significantly higher than all the bone powder products (Figure 1B). 

For the bioaccessibility of phosphorus, the bone powder product CodC showed the highest bioaccessibility of 11%, while the lowest bioaccessibility was observed for the bone powder product HakeE at 1.2% (Figure 1D). The raw cod backbone had a bioaccessibility of phosphorus of 9.7%, which was not significantly different from the bioaccessibility found for the CodC bone powder product (Figure 1D).

The bone powder product CodE showed a significantly higher bioaccessibility of potassium (83%) than HakeE and SalmonE, which had bioaccessibilities of potassium of 64% and 66%, respectively (Figure 1F).

The bone powder product which showed the highest bioaccessibility of calcium was HakeE, at 14%; meanwhile, all other bone powder products had a significantly lower bioaccessibility of calcium. The lowest bioaccessibility was observed for the raw cod backbone, which had a bioaccessibility of calcium of 0.4% For the calcium supplement, a bioaccessibility of calcium of 62% was observed (Figure 1H). 

### 2.3. Bioaccessibility of Proteins from the Different Bone Powder Products and Raw Cod Backbone

**Figure 2:**
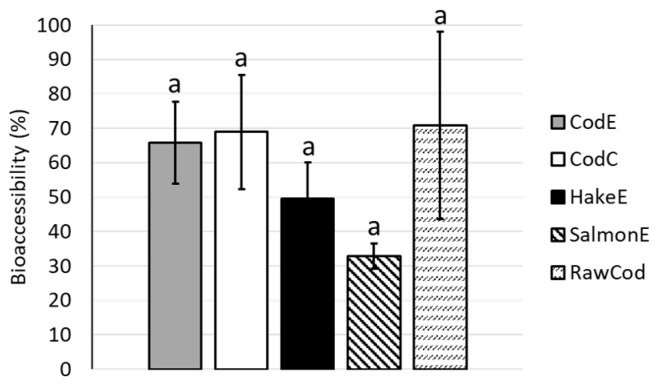
Bioaccessibility of protein (%) in the different bone powder products (CodE, CodC, HakeE, SalmonE) and the raw cod backbone (RawCod). Different letters indicate significant differences (*p*-value < 0.05). Error bars depict the standard deviations. All samples were analysed in triplicate (*n* = 3).

The bioaccessibilities of proteins from the different bone powder products were also assessed and were found to be in the range of 33–71%. No significant differences between the different bone powder products and the raw cod backbone were observed (Figure 2). The lack of significant differences was due to high standard deviations for some of the samples. 

## 3. Discussion

### 3.1. Content of Minerals in the Bone Powder Products and Raw Cod Backbone

The contents of the different minerals in the bone powder products are high and, if bioaccessible, at nutritionally relevant levels, as an intake of 1 g will contribute approximately 10% and 20% of the daily recommended intake of phosphorus (520 mg/day) and calcium (950 mg/day), respectively [16]. 

A previous study showed that alkaline treatment of backbones (Baltic cod and Atlantic salmon) after removal of muscle tissue resulted in a bone powder with a content of 27.79% and 24.92% calcium, 13.40% and 12.50% phosphorus, 0.66% and 0.46% magnesium and 0.030% and 0.027% potassium in the preparations from Baltic cod and Atlantic salmon, respectively. Protein contents were 15% and 18% in the powders obtained from Baltic cod and Atlantic salmon, respectively [17]. Hence, the alkaline treatment of backbone rinsed from muscle tissue resulted in higher contents of calcium and phosphorus, whereas the contents of the minerals magnesium and potassium and proteins were lower. Moreover, calcination (700 °C, 2 h) treatment of fish bones from different species (catfish, tilapia, seabass and tuna) also resulted in higher contents of calcium and phosphorus and lower contents of magnesium and potassium [18]. In contrast, untreated fish bones (cod and salmon) cleaned from muscle tissue showed similar ranges of protein, calcium, phosphorus and magnesium to the bone powder products obtained in the current study, but higher contents of potassium [4]. Hence, the differences observed in the contents of the bone powders are assumed to be ascribed to the differences in the processing of the fish bones and the ratio between fish bones and proteins in the analyzed material. Proteins are degraded in the alkaline and calcination treatments.

### 3.2. Bioaccessibility of Minerals from the Bone Powder Products and Raw Cod Backbone

For the in vitro digestion model (INFOGEST 2.0), the amount of minerals in one gram of bone powder products and calcium supplement digested was approx. 1–5 mg magnesium and 100–350 mg calcium. The results of bioaccessibility for magnesium and calcium in the supplement were 95% and 62%, respectively, while the bioaccessibility of the two minerals from the bone powder products were from 45% up to 74% for magnesium and from 6% up to 14% for calcium. Using a similar analytical approach for the quantification of the minerals but a slightly different digestion model, the bioaccessibility of magnesium and calcium from residues of lime, orange and a mixture of lime and orange [19]; a plant (*Sarcocornia ambigua*) [20]; and biscuits with different compositions of flour and dietary fibres [21] was reported as 30–85%, 65% and 65–78%, respectively, for magnesium, and 33–40%, 3% and 23–55%, respectively, for calcium. Thus, the bioaccessibility of magnesium and calcium in the bone powder products are within the range formerly reported for alternative mineral food products. In the current study, the calcium supplement showed a similarly higher bioaccessibility for magnesium than for calcium, which could indicate that the enzymatic solution used for the digestion does not interfere with the solubility of free magnesium but does for that of free calcium due to proteins [6,21]. The lower bioaccessibility of magnesium and calcium in the current study and other potential food products is thought to rely on other compounds such as proteins, fibers, fatty acids and phytates [6,21].

For the bioaccessibility of the minerals from the bone powder products, no clear difference between the powders was observed (Figure 1). By comparing the two samples, CodC and CodE, we assessed whether the enzyme treatment affected the bioaccessibility. A significant difference between the two samples was only observed for the bioaccessibility of phosphorus, while no significant differences were observed for the bioaccessibility of magnesium, potassium or calcium (Figure 1B,D,F,H). The bioaccessibility of phosphorus was significantly higher after heating (CodC) than after enzyme treatment (CodE). The effect of enzyme treatment on bioaccessibility was tested as it is used in the processing of cod backbone to produce a savoury ingredient, where CodE is the side-stream product generated from the production of the savoury ingredient. This processing step cannot be said to increase the bioaccessibility of minerals but in general did not negatively affect the bioaccessibility of minerals either. 

The bone powder products, HakeE and SalmonE, both only contained the bone fraction of the fish; all meat had been removed from the fraction in the hydrolysis and subsequent sieving process. In contrast, the CodE and CodC bone powders still contained insoluble proteins because the hydrolysates were not sieved to separate the bones from the insoluble proteins before centrifugation. HakeE showed the highest bioaccessibility of magnesium and calcium, while SalmonE showed comparable bioaccessibilties to those of CodE and CodC (Figure 1B,H). There was no clear difference between the bioaccessibilities of phosphorus and potassium from the bone powder products HakeE and SalmonE compared to the bone powder products that contained insoluble proteins from the fish meat, i.e., CodE and CodC (Figure 1D,F). The presence of insoluble proteins from the fish meat in the bone powder products did therefore not in general affect the bioaccessibility of the minerals. 

CodE and CodC bone powder products were produced from cod backbone, while SalmonE was produced from the backbones of salmon and HakeE was made from the bones of undersized whole hake. The results did not suggest that the species from which the products originated from had a significant influence on the bioaccessibility of the minerals from the bone powder products (Figure 1). 

### 3.3. Bioaccessibility of Proteins from the Bone Powder Products and Raw Cod Backbone

The bioaccessibility of protein did not differ between any of the studied bone powder products or the raw cod backbone (Figure 2). Cod backbone contains both soluble and insoluble proteins. The bioaccessible proteins are free amino acids or very short peptides. When processing the bone powder products, the soluble proteins were removed during separation and found in the hydrolysates. Therefore, it could be expected that the bioaccessibility of the proteins in the bone powder products CodE and CodC would be lower than in the raw material (RawCod). However, due to the high standard deviations, no significant differences were observed between any of the samples. 

The amount of protein added to each digest has been shown in other studies to affect the bioaccessibility [22]. In this study, the same amount of sample was weighed in for every digest and, due to the differences in the protein contents of the bone powders (Table 1), different amounts of protein were digested. This may have influenced the bioaccessibility, as has been seen elsewhere [22].

As proteins are primarily absorbed as free amino acids, di- or tri-peptides, it is a limitation of this study that we have not determined these [13]. The DUMAS method was applied in this study and quantified all N that were present in the digesta as particles of <450 nm; we are therefore at risk of overestimating the protein bioaccessibility. 

### 3.4. The Nutritional Relevance of the Bone Powder Products

The nutrient contents of food products provide insight into how a dietary intake could optimally help cover the recommended dietary intake. In this study, in vitro bioaccessibility was assessed for the new potential food ingredients. As seen from Figure 1, the bioaccessibilities of magnesium and calcium from the bone powder products were all significantly lower than for the calcium supplement. This indicates that a consumer has to ingest relatively high amounts of the bone powder products in order to have the same benefit as the calcium supplement, which might not be desirable. Thus, these bone powder products may not be suitable as dietary supplements for minerals. However, a higher bioaccessibility may be expected if the bone powder products are exposed to further processing such as a calcination followed by solubilization in weak acids. In contrast to enzyme hydrolysis, such treatments will solubilise the hydroxyapatite crystals, which are composed of a complex of calcium and phosphorus and insoluble in the gut [23]. 

No clear differences in the bioaccessibilities of the bone powder products were found. Hence, by using the precipitate after hydrolysis (CodE, CodC) and not only fish bones (HakeE and SalmonE), the utilization degree of the side-streams generated in the fish industry can be increased with a minimal generation of new side-streams. Moreover, these bone powder products also had a higher protein concentration. However, some of the minerals tended to have a higher content in the bone powder products with only fish bone (HakeE and SalmonE); using these will also increase the utilization degree for human consumption, but insoluble proteins will still be left-over after sieving of the hydrolysates to obtain the bone fraction. The insoluble protein fraction, mainly composed of non-collagenoues proteins, can be used for fishmeal production. Normally, the cost of raw material and production including enzymes should be considered in the production of new ingredients such as bone powder products. However, the bone powder products evaluated in this study were generated as new side-streams during enzymatic hydrolysis. Thus, the only process added to obtain the mineral powder was drying and milling to obtain full utilization of the entire biomass. Further studies are needed to investigate possible applications of mineral and protein-rich powders for food and feed applications. 

## 4. Materials and Methods

### 4.1. Materials

The bone powder products were generated from different seafood side-streams: cod (*Gadus morhua*) backbone, undersized hake (*Merluccius merluccius*) and salmon (*Salmo salar*) backbone. 

Cod backbone was provided by Royal Greenland and is a side-stream generated in cod filleting in Maniitsoq (Greenland). The backbone was collected from the waste belt of a fileting machine. Freshly collected cod backbones were frozen and shipped under freezing conditions from the factory in Greenland to Denmark (shipping approx. 1 month, −20 °C). At DTU, the cod backbones were stored at ≤−40 °C until the enzymatic hydrolysis experiments were carried out.

Undersized hakes, as a model of discard from the Basque fleet, were collected in Ondarroa Harbour, Spain, from a bottom trawler in October 2019. Once landed, the samples were directly brought to AZTI’s facilities, packed in vacuum in small quantities and stored frozen (−18 °C) until used. Fish were stored and processed whole, not gutted.

Salmon backbones, a side-stream from salmon filleting, were collected from Barna, a fishmeal and oil producer from Bermeo, Spain. Backbones were collected separately from the filleting plant, preserved and transported refrigerated to AZTI’s facilities. Salmon backbones were then frozen and stored at −18 °C before being processed. 

Enzymes used for the enzymatic hydrolysis of the different fish side-streams were provided by Novozymes (Bagsværd, Denmark).

### 4.2. Samples

Three of the samples were a co-product generated from enzymatic hydrolyses of different seafood side-streams, i.e., the precipitate after hydrolysis. One of the samples as a reference for the enzymatic hydrolysis (without enzyme added) and one cod backbone before enzymatic/heat treatment (RawCod).

CodE: This sample was derived from the enzymatic production of savoury ingredients from minced cod backbone. The treatment to produce savoury ingredients involved no pH adjustment and no inactivation of endogenous enzymes prior to the hydrolysis. The enzymes used were Alcalase, Exomixture (Protana Prime, Novozymes, Bagsværd, Denmark) and Glutaminase (Protana UBoost, Novozymes, Bagsværd, Denmark). The sample was diluted with water to a protein concentration of 12%, and each enzyme was added in a concentration of 1% based on the protein concentration in the sample. The hydrolysis was carried out at 55 °C for 6 h followed by inactivation of the enzymes (90 °C, 15 min) and centrifugation to separate hydrolysate (supernatant) and precipitate (Sorval RC 6+ centrifuge (Thermo Fisher Scientific, Waltham, MA, USA), FiberLite® F10–6x500y rotor, 14,260× *g*, 20 min). The precipitate was collected for the study, dried in an oven (50 °C, 1–2 days), homogenized using a mortar and pistol and stored at −40 °C until further analysis.

CodC: This sample was derived in similar way to CodE though without enzyme addition, i.e., only heat treatment, as a control of the efficiency of the enzyme in the production of savoury ingredients.

HakeE: Hake side-streams were mixed with water in a ratio of 1:1, and enzyme was added. The hydrolysis was carried out between 50 °C and 60 °C for 3 h, whereafter the enzymes were inactivated (90 °C, 15 min). The bones were separated by screening the content of the hydrolysis reactor through a 1.6 mm sieve. After that, bones were gently dried in a static oven for 2 h at 80 °C. Then, the bones were milled in an Ultra Centrifugal Mill (Retsch ZM 100, Haan, Germany) at 18,000 r.p.m. mill with a sieve of 1 mm. Bones were vacuum-packed and stored in a dry and cool place. The bone powder product used was obtained as a pooled sample obtained from the use of different combinations of endo and exopeptidases, and the conditions are described in Iñarra et al., 2023 [3]. The bone powder products were pooled to one as there were no significant differences in the compositions of the bone fraction obtained. 

SalmonE: Salmon bone powders were pooled from an experiment using different combinations of endo and exopeptidases; the aim was the production of savoury compounds following a similar procedure as for HakeE. 

RawCod: This sample was minced cod backbone without enzymatic treatment and/or heat treatment, i.e., the starting material for samples CodE and CodC. The dry matter content in the cod backbone was 23.2 ± 1.8%.

Table 2 gives an overview of the amount of pellet and bone fractions generated per 100 g of raw material in the enzymatic hydrolysis and the dry matter.

The supplement (Kalkwas, Lekaform) was bought at the local supermarket. Before analysis, 10 tablets were weighed off and homogenised to a fine powder using a mortar. The supplement powder was stored at −20 °C in darkness in a 15 mL Sarstedt tube overflown with nitrogen. 

### 4.3. Mineral Analysis

The contents of magnesium (Mg), phosphorus (P), potassium (K) and calcium (Ca) were determined via inductively coupled plasma mass spectrometry (Thermo iCAPQ ICPMS) following microwave-assisted digestion (Multiwave 7000, Anton Paar, Graz, Austria) using concentrated nitric acid (SPS Science, Paris, France) for the bone powder products and raw cod backbone. The digest samples from the INFOGEST 2.0 were diluted with 2% HNO_3_ in MiliQ water prior to analysis. Quantification was performed by external calibration with internal standardization, using Rh as an internal standard. All calibration standards were prepared from certified stock solutions (SPS Science). The certified reference material (DORM-5 fish protein, NRC, Ottawa, ON, Canada) was analysed together with the samples used for quality assurance of the analytical results. The obtained results were in good agreement with certified values (recoveries were in the range of 92–112% of certified value for all elements). All the samples were diluted 500× or 1000× prior to analysis. 

### 4.4. Dry Matter Determination

The dry matter was measured gravimetrically using an oven to dry the bone powder products (105 °C, 20–24 h) obtained. 

### 4.5. Protein Analysis

The protein content in the bone powder products was measured by DUMAS using a conversion factor of 5.58 [24].

### 4.6. In Vitro Digestion (INFOGEST 2.0)

The digestion followed the procedure described in detail elsewhere [14]. In short, the samples were subjected to sequential oral, gastric and intestinal digestion using 0.5 g of sample and 0.5 g of water. After the intestinal phase, the samples were centrifuged at 5000× *g* at 37 °C for 1 h and thereafter filtered through a 0.45 µm syringe filter. The final digesta after filtration was stored at −20 °C until further analysis. 

### 4.7. Statistical Analysis

One-way ANOVA and Tukey’s range test were used to assess whether there was a significant difference (*p*-value < 0.05) between the different bioaccessibilities for the minerals and protein. All statistical work was performed using Excel (Microsoft^®^ Excel^®^ 2016) and Rstudio (Version, 1.2.1335).

## 5. Conclusions

The contents of minerals and protein in the bone powder products showed bioaccessibilities of 1.2–83% and 33–69%, respectively. There were no clear differences in the bioaccessibilities of either minerals or proteins for the different bone powder products, and it can hereby be concluded that the processing differences between the bone powder products had no significant effect on their bioaccessibility. The bioaccessibility for magnesium and calcium were within the reported range of other alternative mineral food products, such as residues from lime, orange and biscuits. 

The bone powder products had a high content of minerals (e.g., calcium 15.7–21.6 g/100 g) and also a good content of proteins (19–41%). Therefore, the new side-streams generated from enzymatic hydrolysis can in the future be used as a potential mineral and protein-rich powder for the food sector, due to the availability of bioaccessible proteins and minerals. Moreover, using these bone powder products in food and feed applications increases the utilization degree of fish in the fish industry.

## Figures and Tables

**Table 1:** Content of the minerals (mg/100 g) magnesium (Mg), phosphorus (P), potassium (K) and calcium (Ca) as well as proteins (%) in the calcium supplement (Ca supplement), the four bone powder products obtained after enzymatic hydrolysis (CodE, CodC, HakeE, SalmonE) and the untreated cod backbone (RawCod). We used 2–5 replicates for the minerals and none for the proteins (*n* = 1). For detailed sample description refer to Section 4.2. Different letters in superscript indicate significant differences between samples within each mineral.

Food Matrix	Minerals	Mg	P	K	Ca	Protein	
	*n*	mg/100 g				*n*	(%)
Ca supplement	2	127	1.7	8.3	33,216	1	-
CodE	5	310 ^B^ ± 10	8200 ^C^ ± 500	490 ^B^ ± 10	15,700 ^C^ ± 900	1	41
CodC	3	240 ^C^ ± 10	5800 ^D^ ± 100	580 ^A^ ± 10	10,700 ^D^ ± 200	1	54
HakeE	3	460 ^A^ ± 10	11100 ^A^ ± 200	310 ^C^ ± 10	21,600 ^A^ ± 800	1	28
SalmonE	3	290 ^B^ ± 10	930 ^B^ ± 400	240 ^D^ ± 10	17,400 ^B^ ± 800	1	32
RawCod	3	40 ^D^ ± 10	1000 ^E^ ± 300	240 ^D^ ± 10	1900 ^E^ ± 600	1	19

**Table 2:** Overview of the mass obtained for the different treatments given as pellet or bone fraction per mass of side-streams and the dry matter (%) content (average and standard deviation (*n* = 2–4)).

Samples	Mass (g/100 g Side-Stream) *		Dry Matter (%)
	Pellet Fraction	Bone Fraction	
CodE	14.5 ± 3.8	-	97.6 ± 0.2
CodC	19.4 ± 3.8	-	98.0 ± 0.3
SalmonE	-	25.5 ± 2.6	91.6 ± 2.9
HakeE	-	9.4 ± 0.7	97.3 ± 0.0

* Pellet fraction is the residual dried mass after enzyme hydrolysis when supernatant (soluble proteins, peptides, amino acids) is removed, i.e., insoluble proteins, peptides and bone fraction. Whereas, for the bone fraction, insoluble proteins are removed, and bones were retained during reactor discharge in a sieve without drying.

## Data Availability

The data presented in this study are available on request from the corresponding authors.
